# Increased sympathetic nerve activity and reduced cardiac baroreflex sensitivity in rheumatoid arthritis

**DOI:** 10.1113/JP272944

**Published:** 2016-10-24

**Authors:** Ahmed M. Adlan, Julian F. R. Paton, Gregory Y. H. Lip, George D. Kitas, James P. Fisher

**Affiliations:** ^1^ College of Life & Environmental SciencesUniversity of BirminghamBirminghamUK; ^2^ School of Physiology, Pharmacology & Neuroscience, Biomedical SciencesUniversity of BristolBristolUK; ^3^ University of Birmingham Centre of Cardiovascular SciencesCity HospitalBirminghamUK; ^4^ Department of Rheumatology, Dudley Group NHS Foundation TrustRussells Hall HospitalDudleyWest MidlandsUK

**Keywords:** autonomic nervous system, cytokine, inflammation, pain

## Abstract

**Key points:**

- Rheumatoid arthritis (RA) is a chronic inflammatory condition associated with an increased risk of cardiovascular mortality.
- Increased sympathetic nerve activity and reduced cardiac baroreflex sensitivity heighten cardiovascular risk, althogh whether such autonomic dysfunction is present in RA is not known.
- In the present study, we observed an increased sympathetic nerve activity and reduced cardiac baroreflex sensitivity in patients with RA compared to matched controls.
- Pain was positively correlated with sympathetic nerve activity and negatively correlated with cardiac baroreflex sensitivity.
- The pattern of autonomic dysfunction that we describe may help to explain the increased cardiovascular risk in RA, and raises the possibility that optimizing pain management may resolve autonomic dysfunction in RA.

**Abstract:**

Rheumatoid arthritis (RA) is a chronic inflammatory condition associated with increased cardiovascular morbidity/mortality and an incompletely understood pathophysiology. In animal studies, central and blood borne inflammatory cytokines that can be elevated in RA evoke pathogenic increases in sympathetic activity and reductions in baroreflex sensitivity (BRS). We hypothesized that muscle sympathetic nerve activity (MSNA) was increased and BRS decreased in RA. MSNA, blood pressure and heart rate (HR) were recorded in age‐ and sex‐matched RA‐normotensive (*n = *13), RA‐hypertensive patients (RA‐HTN; *n = *17), normotensive (NC; *n = *17) and hypertensive controls (HTN; *n = *16). BRS was determined using the modified Oxford technique. Inflammation and pain were determined using serum high sensitivity C‐reactive protein (hs‐CRP) and a visual analogue scale (VAS), respectively. MSNA was elevated similarly in RA, RA‐HTN and HTN patients (32 ± 9, 35 ± 14, 37 ± 8 bursts min^–1^) compared to NC (22 ± 9 bursts min^–1^; *P = *0.004). Sympathetic BRS was similar between groups (*P = *0.927), whereas cardiac BRS (cBRS) was reduced in RA, RA‐HTN and HTN patients [5(3–8), 4 (2–7), 6 (4–9) ms mmHg^–1^] compared to NC [11 (8–15) ms mmHg^–1^; *P = *0.002]. HR was independently associated with hs‐CRP. Increased MSNA and reduced cBRS were associated with hs‐CRP although confounded in multivariable analysis. VAS was independently associated with MSNA burst frequency, cBRS and HR. We provide the first evidence for heightened sympathetic outflow and reduced cBRS in RA that can be independent of hypertension. In RA patients, reported pain was positively correlated with MSNA and negatively correlated with cBRS. Future studies should assess whether therapies to ameliorate pain and inflammation in RA restores autonomic balance and reduces cardiovascular events.

## Key points

- Rheumatoid arthritis (RA) is a chronic inflammatory condition associated with an increased risk of cardiovascular mortality.
- Increased sympathetic nerve activity and reduced cardiac baroreflex sensitivity heighten cardiovascular risk, althogh whether such autonomic dysfunction is present in RA is not known.
- In the present study, we observed an increased sympathetic nerve activity and reduced cardiac baroreflex sensitivity in patients with RA compared to matched controls.
- Pain was positively correlated with sympathetic nerve activity and negatively correlated with cardiac baroreflex sensitivity.
- The pattern of autonomic dysfunction that we describe may help to explain the increased cardiovascular risk in RA, and raises the possibility that optimizing pain management may resolve autonomic dysfunction in RA.

**Abbreviations**
BMI: body mass index
BP: blood pressure
BRS: baroreflex sensitivity
cBRS: cardiac baroreflex sensitivity
HR: heart rate
hs‐CRP: high sensitivity C‐reactive protein
HTN: hypertensive
IL: interleukin
MOT: modified Oxford technique
MSNA: muscle sympathetic nerve activity
NC: normotensive controls
RA: rheumatoid arthritis
RA‐HTN: rheumatoid arthritis hypertensive
TNF: tumour necrosis factor
VAS: visual analogue scale

## Introduction

Rheumatoid arthritis (RA) is a chronic inflammatory condition associated with increased risk of cardiovascular mortality and myocardial infarction, as well as an increased prevalence of hypertension and cardiac arrhythmias (Maradit‐Kremers *et al*. 2005; Arosio *et al*. 2007; Levy *et al*. 2008; Panoulas *et al*. 2008). Traditional cardiovascular risk factors are present in RA patients but do not wholly account for the increased cardiovascular risk (Maradit‐Kremers *et al*. 2005) and hence novel mechanisms are sought (Amaya‐Amaya *et al*. 2013; Panoulas *et al*. 2014).

A heightened central sympathetic outflow to the heart and/or vasculature has been identified in numerous cardiovascular diseases (Fisher & Paton, 2012) and is associated with the pathogenesis of hypertension (Fisher & Paton, 2012), cardiac arrhythmias (Lown & Verrier, 1976) and increased mortality (Cohn *et al*. 1984; Barretto *et al*. 2009). Reciprocal links between inflammation and the sympathetic nervous system have been identified (Niijima *et al*. 1991; Zhang *et al*. 2003; Helwig *et al*. 2008). Central sympatholytic agents have anti‐inflammatory actions in human hypertension (Poyhonen‐Alho *et al*. 2008), whereas the infusion of inflammatory cytokines known to be elevated in RA patients, such as interleukin (IL)‐6 (Helwig *et al*. 2008), tumour necrosis factor (TNF)‐α (Zhang *et al*. 2003) and IL‐1β (Niijima *et al*. 1991), increases sympathetic nerve activity in rats. Thus, the chronic activation of inflammatory cytokines in RA may elicit deleterious increases in sympathetic neural outflow that further promote activation of pro‐inflammatory cytokines. Furthermore, microinjection of IL‐6 into the nucleus of the solitary tract, a key central regulatory cardiovascular site, is reported to reduce cardiac baroreflex sensitivity (BRS) (Takagishi *et al*. 2010). Impaired BRS is present in hypertension (Gribbin *et al*. 1971), heart failure (Ferguson *et al*. 1992) and coronary artery disease (De Ferrari *et al*. 2007), and is reported to predict reduced survival following myocardial infarction (La Rovere *et al*. 1998; De Ferrari *et al*. 2007) and in patients with heart failure (La Rovere *et al*. 2009). Depressed BRS may potentially contribute to the recognized increased cardiovascular risk in RA (Maradit‐Kremers *et al*. 2005; Levy *et al*. 2008) by precipitating further increases in central sympathetic outflow and reducing cardiac electrical stability.

Whether sympathetic activity is increased and BRS is reduced in RA is presently equivocal, partly as a result of the indirect methods of assessment used to examine this (Adlan *et al*. 2014). Elevations in plasma noradrenaline have been reported in RA patients (Vlcek *et al*. 2008), although it is not clear whether this is a consequence of increased central sympathetic outflow, enhanced release from peripheral adrenergic stores or altered local reuptake mechanisms (Esler *et al*. 1990). Such limitations are overcome by the microneurography technique, which provides a direct assessment of sympathetic nerve activity to the skeletal muscle vasculature (MSNA), although such measures have not been undertaken previously in RA. It is also not known whether baroreflex regulation of MSNA is altered in RA. Although a reduction in cardiac BRS has been suggested in RA patients (Aydemir *et al*. 2010), baroreflex control of the heart and vasculature do not always function in a parallel fashion (Rudas *et al*. 1999).

We aimed to test the hypothesis that central sympathetic outflow is elevated in RA patients and is associated with elevated circulating concentrations of inflammatory cytokines. We made the first direct recordings of MSNA in patients with RA. Additionally, we examined whether the sensitivity of baroreflex control of the heart and MSNA are reduced in RA patients and associated with an elevated concentration of circulating inflammatory cytokines. Hypertension control groups (RA and non‐RA) were included to attempt and control for the effect of medications and comorbidities.

## Methods

### Participants

Thirty patients with a clinical diagnosis of RA based on the 1987 American College of Rheumatology criteria (Arnett *et al*. 1988) were recruited from the rheumatology clinics at Russells Hall Hospital, Dudley, UK, and Sandwell General Hospital, West Bromwich, UK. The 1987 American College of Rheumatology criteria allows for classification of RA when four of its seven criteria (morning stiffness, arthritis of three or more joint areas, arthritis of hand joints, symmetric arthritis, rheumatoid nodules, positive serum rheumatoid factor and radiographic changes) are present (morning stiffness, arthritis and rheumatoid nodules need to be present for at least 6 weeks). Exclusion criteria included: age <18 or >75 years, atrial fibrillation or other heart rhythm disorder, significant valvular disease, coronary artery disease, diabetes, ischaemic stroke, chronic renal failure, liver impairment, hormone replacement therapy, and those who are pregnant or who might be pregnant. In addition, 33 age‐, sex‐ and body mass index (BMI)‐matched control participants were recruited from the hospitals and surrounding areas. Normotensive control (NC) participants were free from major illnesses, whereas hypertensive controls (HTN) either had a prior diagnosis of hypertension or blood pressure (BP) ≥140/90 mmHg. Ethical approval was obtained by the local Research Ethics Committee (National Research and Ethics Service Committee West Midlands – Edgbaston, 11/WM/0298). Written informed consent was received from participants prior to inclusion in the study, in accordance with the *Declaration of Helsinki*.

### Experimental protocol

Participants were studied according to an observational, case–control study that included four groups: 13 RA patients, 17 RA‐HTN patients, 16 HTN and 17 NC. Participants attended the research laboratory at 09.00 h after an overnight fast (from food, caffeine, alcohol and nicotine). All medications were withheld on the morning of testing. A detailed clinical history was obtained and physical examination was performed in RA patients to count the number of swollen and tender joints aiming to determine the disease activity score C‐reactive protein (DAS28‐CRP) (Wells *et al*. 2009). A visual analogue scale (VAS) was used as a measure of patient‐reported pain (Huskisson, 1974). Height and weight was measured, and the BMI was determined (weight/height^2^). All subsequent measurements were performed under uniform conditions in a temperature‐controlled room when the participants were resting quietly in the supine position.

Following instrumentation for heart rate (HR), BP, leg blood flow and MSNA as described below, an i.v. catheter was inserted into a superficial vein in the antecubital fossa for blood sampling and injections. The experimental protocol involved a 10 min baseline period followed by sequential infusion of sodium nitroprusside (100 μg of sodium nitroprusside) and phenylephrine (150 μg of phenylephrine) 1 min later (modified Oxford technique; MOT) (Rudas *et al*. 1999).

### Measurements

HR was continuously recorded using a lead II electrocardiogram (ECG, BioAmp; ADInstruments, Bella Vista, Australia). Beat‐to‐beat BP was recorded using finger photoplethysmography (Portapres; Finapres Medical Systems, Amsterdam, The Netherlands) and was calibrated with brachial BP recordings using an automated sphygmomanometer (Omron 705IT; Omron Corporation, Hoopddorp, The Netherlands). Multi‐unit recordings of MSNA were obtained (FE185 NeuroAmp EX; ADInstruments) from the peroneal nerve using tungsten microelectrodes (200 μm, 1–3 μm uninsulated tip; UNA32F2S; FHC, Bowdoin, ME, USA). Electrical stimulation on the skin surface was used for nerve mapping, and a reference electrode was inserted s.c. 2–3 cm away from the recording electrode, which was inserted into the nerve fascicle. The neural signals were amplified, filtered (100 Hz low pass, 2000 Hz high pass, 60 Hz notch), rectified and integrated (absolute value, time constant decay 0.1 s) to obtain a mean voltage of sympathetic nerve activity. MSNA was confirmed by: listening to the amplified signal on speakers; observing the characteristic bursting pattern on a computer screen; palpating the skin and muscle fibres; and performing a breath hold (Wallin & Fagius, 1988; Grassi & Esler, 1999). Leg blood flow was measured using venous occlusion strain gauge plethysmography in accordance with standard methods (Hokanson EC‐6 plethysmograph; DE Hokanson, Bellevue, WA, USA) (Joyner *et al*. 2001). A lightweight indium‐in‐silastic strain gauge was positioned around the right calf at the point of greatest circumference. The length of the strain gauge was 2 cm less than the widest girth of the calf. Cuffs were placed around the right ankle and inflated to a pressure of 200 mmHg and maintained for 1 min to achieve arterial occlusion. Sixty seconds later, cuffs placed around the thigh were rapidly inflated to 50 mmHg (Hokanson E20 rapid cuff inflator and AG101 air source; Hokanson, Bellevue, WA, USA) to evoke venous occlusions. Venous occlusion was repeated three times during 1 min, with the thigh cuffs inflated for 5 s and then deflated for 10 s each time.

### Data analysis

Data were acquired using the Powerlab 16/35 data acquisition system and Labchart Pro software (ADInstruments) and analysed offline. Sympathetic bursts were identified by a single observer (AMA) using a semi‐automated scoring system created using Spike 2 (Cambridge Electronic Design, Cambridge, UK). MSNA burst frequency (bursts min^–1^) and incidence [bursts (100 heart beats)^–1^] was determined. Assessment of cardiac BRS was determined during the phenylephrine induced rise in BP (MOT) (Rudas *et al*. 1999; Studinger *et al*. 2007). Analysis was performed from the first concordant change in systolic BP and RR‐interval until they were discordant. To account for baroreflex delays, systolic BP was associated with concurrent (resting RR‐interval >800 ms) or subsequent RR‐intervals (RR‐interval between 500 and 800 ms) (Pickering & Davies, 1973; Eckberg & Eckberg, 1982). Respiratory related variations in RR‐interval were accounted for by averaging RR‐intervals over 3 mmHg pressure bins (Ebert, 1990). Assessment of baroreflex control of MSNA was determined using linear regression [diastolic BP (DBP) *vs*. MSNA burst activity] during the phenylephrine induced rise in BP (MOT) (Hart *et al*. 2010). Briefly, each cardiac cycle was scored according to the presence or absence of a sympathetic burst. Data were binned according to the DBP of the previous cardiac cycle (3 mmHg bins). Linear regression analysis was then performed on the relationship between the mean of each DBP bin and MSNA burst incidence (bursts/100 heart beats). All data were weighted for the number of cardiac cycles in each DBP bin (Hart *et al*. 2010). We used the slope of the linear regression [(bursts/100 heart beats)/mmHg] as an index of sympathetic BRS. Some patients were unsuitable for microneurography (e.g. discomfort laying still for an extended period or declined microneurography) and it was not possible to obtain sufficiently high quality MSNA recordings in all patients; thus, these results were omitted from the MSNA analyses. Participant numbers are stated as appropriate.

### Blood sampling

Blood samples for inflammatory markers were centrifuged immediately and the plasma stored at –80°C. Commercially available enzyme‐linked immunosorbent assay kits were used to determine high sensitivity C‐reactive protein (hs‐CRP) (MP Biomedicals, Santa Ana, CA, USA) and cytokines (IL‐6, TNF‐α, IL‐10; BioSupply UK, Bradford, UK). The intra‐ and inter‐assay coefficients of variations were 7.5% and 4.1%, respectively, for hs‐CRP; 4.9% and 6.0% for IL‐6; 8.5% and 9.8% for TNF‐α; and 6.8% and 7.5% for IL‐10. Local routine clinical laboratories were used to analyse full blood count, renal function, fasting lipid profile and fasting glucose.

### Statistical analysis

Statistical analysis was performed using SPSS, version 19 (IBM Corp., Armonk, NY, USA). Continuous variables were tested for normality using the Kolmogorov–Smirnov test. Non‐parametric data were (naturally) logarithmically transformed. Group differences were assessed using ANOVA with a least significance difference *post hoc* test for continuous variables, as well as Pearson's chi‐squared test for categorical data. Differences between the RA normotensive and RA‐HTN groups were assessed using an independent *t* test. Associations between autonomic parameters and inflammation were assessed before (Pearson's product/Spearman's rank correlation coefficient) and after adjustment for potential confounders. Data are expressed as the mean ± SD for parametric data; geometric mean (95% confidence interval) for non‐parametric data; and frequency (percentages) for categorical variables. *P* < 0.05 was considered statistically significant.

## Results

Baseline participant characteristics are presented in Table 1. Aside from hypertension, there were no significant differences in other cardiovascular risk factors between the groups. Compared to the RA and NC groups, there was a higher prevalence of osteoarthritis (*P = *0.003) and statin therapy (*P = *0.032) in the RA‐HTN and HTN groups. The RA and RA‐HTN groups had a higher prevalence of proton pump inhibitor (*P = *0.016) and folic acid (*P < *0.001) therapy, whereas calcium/vitamin D supplementation tended to be highest in the RA‐HTN group (*P = *0.06). Similar anti‐hypertensive agent use was noted in the RA‐HTN and HTN groups. A small number of participants were smokers (*P = *0.064 between groups), although no use of other nicotine containing products was reported by any participant.

**Table 1:** Subject characteristics

	RA	RA‐HTN	NC	HTN	
	(*n* = 13)	(*n* = 17)	(*n* = 17)	(*n* = 16)	*P*
Age (years)	55.9 ± 11.7	60.5 ± 9.6	53.6 ± 13.0	59.6 ± 10.1	0.257
Female, *n* (%)	8 (62)	12 (71)	10 (59)	11 (69)	0.876
Body mass index, (kg m^–2^)	27.8	29.6	26.4	25.8	0.130
	(25.4–30.4)	(26.4–33.3)	(23.8–29.2)	(24.5–27.2)	
Total cholesterol (mmol L^–1^)	4.8 ± 1.1	5.1 ± 1.0	5.1 ± 0.9	5.1 ± 1.0	0.804
Triglycerides (mmol L^–1^)	1.1 (0.9–1.5)	1.0 (0.8–1.4)	1.1 (0.8–1.3)	1.1 (0.9–1.3)	0.968
HDL (mmol L^–1^)	1.3 (1.1–1.5)	1.5 (1.3–1.8)	1.4 (1.2–1.6)	1.4 (1.2–1.7)	0.498
LDL (mmol L^–1^)^a^	2.9 ± 0.9	2.9 ± 0.9	3.1 ± 0.8	3.0 ± 0.9	0.866
eGFR (ml min^–1^ 1.73 m^–2^)	100.4 ± 20.3	89.9 ± 14.6	88.0 ± 19.6	83.5 ± 17.9	0.099
Haemoglobin (g L^–1^)	126 ± 12^*^, ^†^	133 ± 14	138 ± 10	138 ± 12	0.019
Smoking, *n* (%)	4 (31)	1 (6)	3 (18)	0	0.064
Osteoarthritis, n (%)	2 (15)	9 (53)	0	4 (25)	0.003
Anti‐hypertensive agent, *n* (%)	–	7 (41)	–	11 (69)	0.112
ACEi or ARB, *n* (%)	–	5 (29)	–	5 (31)	0.909
Calcium channel blocker, *n* (%)	–	4 (24)	–	7 (44)	0.218
Thiazide, *n* (%)	–	2 (12)	–	5 (31)	0.171
β‐blocker, *n* (%)	–	2 (12)	–	1 (6)	0.582
α‐blocker, *n* (%)	–	0	–	2 (13)	0.133
Aspirin, *n* (%)	1 (8)	1 (6)	0	1 (6)	0.748
Statin, *n* (%)	1 (8)	6 (35)	0	4 (25)	0.032
Proton pump inhibitor, *n* (%)	4 (31)	6 (35)	0	1 (6)	0.016
Folic acid, *n* (%)	8 (62)	12 (71)	0	0	<0.001
Adcal D3, *n* (%)	2 (15)	5 (29)	1 (6)	0	0.060

Values are expressed as the mean ± SD (parametric) for continuous variables and frequency (percentage) for discrete variables. Non‐parametric data were (natural) log transformed and are displayed as the geometric mean (95% confidence intervals). One‐way ANOVA with a least significance difference *post hoc* test. Pearson's chi‐square test for categorical data. Significance: *P* ≤ 0.05. *Post hoc*: *P* ≤ 0.05.

^*^ *P* ≤ 0.05 *vs*. HC

^†^ *P* ≤ 0.05 *vs*. RA‐HTN.

^a^Calculated using the Friedewald formula.

ACEi, angiotensin‐converting enzyme inhibitor; ARB, angiotensin‐renin blocker; CVD, cardiovascular disease; eGFR, estimated glomerular filtration rate; HDL, high‐density lipoprotein; LDL, low‐density lipoprotein. Adcal D3 is a calcium carbonate, vitamin D3 tablet.

RA disease‐related characteristics are presented in Table 2. The RA normotensive and RA‐HTN groups had similar disease duration, sero‐positivity and RA drug therapy. A greater number of swollen joints (*P = *0.045) and a trend for higher disease activity (DAS28‐CRP, *P = *0.063) were seen in the RA‐HTN group compared to the RA group.

**Table 2:** Rheumatoid arthritis‐related characteristics

	RA	RA‐HTN	
	(*n* = 13)	(*n* = 17)	*P*
RA duration (years)	7.6 (4.6–12.4)	5.5 (2.9–10.5)	0.443
RF positive, *n* (%)	9 (69)	10 (59)	0.768
Swollen joints, *n* (%)	5.2 (2.9–9.0)	2.6 (1.6–4.0)	0.045
Tender joints, *n* (%)	6.7 (2.8–14.7)	3.5 (1.7–6.7)	0.194
DAS28‐CRP	4.8 ± 1.9	3.7 ± 1.2	0.063
Disease activity, *n* (%)			
Remission	4 (31)	5 (31)	0.088
Low disease activity	3 (23)	9 (56)	
High disease activity	6 (46)	2 (13)	
VAS, %	36.8 (21.7–62.0)	12.9 (5.3–30.1)	0.047
DMARD, *n* (%)	11 (85)	15 (88)	0.773
Number of DMARDs	1.8 (1.2–2.4)	1.8 (1.3–2.2)	0.850
Methotrexate, *n* (%)	8 (62)	12 (71)	0.602
Hydroxychloroquine, *n* (%)	5 (38)	10 (59)	0.269
Sulfasalazine, *n* (%)	4 (31)	3 (18)	0.400
Leflunomide, *n* (%)	2 (15)	1 (6)	0.390
Glucocorticoid, *n* (%)	1 (8)	6 (35)	0.077
Prednisolone, *n* (%)	1 (8)	4 (24)	0.249
Prednisolone dose (mg)	3	5.3 ± 3	0.534
Intramuscular, *n* (%)	0	1 (6)	0.374
Intra‐articular, *n* (%)	0	1 (6)	0.374
NSAID, *n* (%)	5 (38)	6 (35)	0.858
Opioid, *n* (%)	6 (46)	7 (41)	0.785
Weak, *n* (%)	5 (38)	7 (41)	0.880
Strong, *n* (%)	1 (8)	0	0.245
Biological agent, *n* (%)	4 (31)	3 (18)	0.400
TNF‐α inhibitor, *n* (%)	4 (31)	1 (6)	0.070
Certolizumab, *n* (%)	2 (15)	0	0.094
Etanercept, *n* (%)	2 (15)	0	0.094
Golilumab, *n* (%)	0	1 (6)	0.374
Rituximab, *n* (%)	0	2 (12)	0.201

Values expressed as the mean ± SD for continuous variables (parametric) and frequency (percentage) for discrete variables. Non‐parametric data were (natural) log transformed and are displayed as the geometric mean (95% confidence intervals). Statistical differences were tested using an independent *t* test for continuous variables and Pearson's chi‐square test for categorical data. Significance: *P* ≤ 0.05.

DAS, disease activity score; DMARD, disease modifying anti‐rheumatic drug; NSAID, non‐steroidal anti‐inflammatory drug; RF, rheumatoid factor.

### Haemodynamic parameters

**Figure 1:**
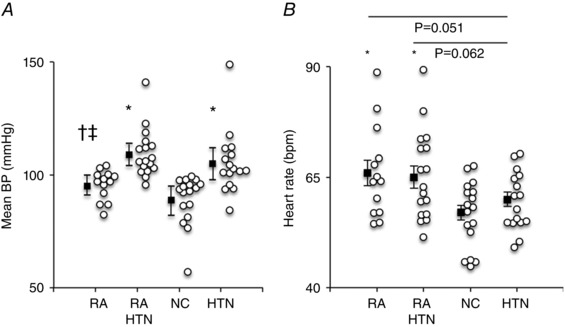
BP and HR Box and whisker plots showing mean blood pressure (*A*, geometric mean and 95% confidence intervals) and heart rate (*B*, mean ± SEM) in the RA, RA‐HTN, NC and HTN groups. Group (black squares) and individual (white circles) data are shown. Overall effect: *P < *0.05. *Post hoc*: ^*^ *P < *0.05 *vs*. NC, ^†^ *P < *0.05 *vs*. HTN, ^‡^ *P < *0.05 *vs*. RA‐HTN. *A* and *B*, RA, *n = *13; RA‐HTN, *n = *17; NC, *n = *17; HTN, *n = *16.

**Figure 2:**
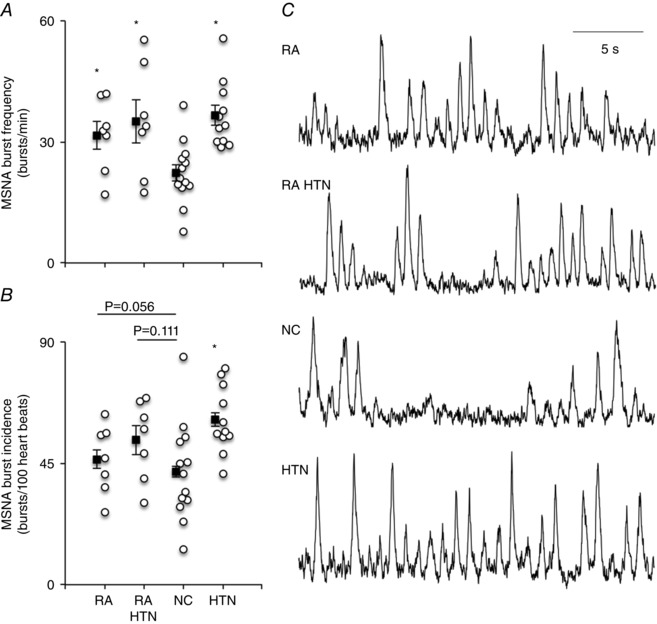
MSNA Box and whisker plots showing group mean ± SEM data for MSNA burst frequency (*A*) and MSNA burst incidence (*B*), as well as original sympathetic neurograms showing MSNA (*C*) in representative individuals from the RA, RA‐HTN, NC and HTN groups. Group (black squares) and individual (white circles) data are shown. Overall effect: *P < *0.05. *Post hoc*: ^*^ *P < *0.05 *vs*. NC. *A* and *B*, RA, *n = *7; RA‐HTN, *n = *7, NC, *n = *13; HTN, *n = *11.

Mean BP was higher in the HTN and RA‐HTN groups compared to the RA and NC groups (*P < *0.001), with no significant difference between the RA‐HTN and HTN groups, or between the RA and NC groups (systolic/diastolic BP RA 129 ± 10/79 ± 6, RA‐HTN 154 ± 18/87 ± 10, HTN 147 ± 25/84 ± 11, NC 123 ± 10/75 ± 6 mmHg; *P < *0.001; *post hoc* analysis, *P < *0.05 for RA *vs*. RA‐HTN and HTN, NC *vs*. RA‐HTN and HTN; diastolic BP RA *vs*. HTN, *P = *0.095) (Fig. 1). Resting HR was higher in the RA and RA‐HTN groups compared to the NC and HTN groups (*P = *0.008). MSNA (burst frequency) was higher in the RA, RA‐HTN and HTN groups compared to the NC group (*P = *0.004) (Fig. 2). When adjusted for HR, there was a significant difference in MSNA (burst incidence) between the groups (*P = *0.029). *Post hoc* analysis showed significantly higher MSNA burst incidence in the HTN group compared to the NC group; and trends for an elevation in the RA‐HTN *vs*. NC groups (*P = *0.111) and between the RA and HTN groups (*P = *0.056) (Fig. 1). Leg blood flow was significantly higher in the RA and RA‐HTN groups compared to the NC group and tended to be higher than in the HTN group (geometric mean, 95% CI, RA 2.0, 1.5–2.6; RA‐HTN 2.0, 1.4–2.8; NC 1.2, 0.9–1.7; HTN 1.4, 1.0–1.8 ml 100^–1^ ml min^–1^; *P = *0.047; *post hoc* analysis RA *vs*. HTN, *P = *0.101, RA‐HTN *vs*. HTN, *P = *0.086). However, leg vascular conductance was similar between all groups (RA 21, 15–27; RA‐HTN 18, 12–26; NC 14, 10–19; HTN 13, 10–17 arbitrary units; *P = *0.148).

### Baroreflex sensitivity

**Figure 3:**
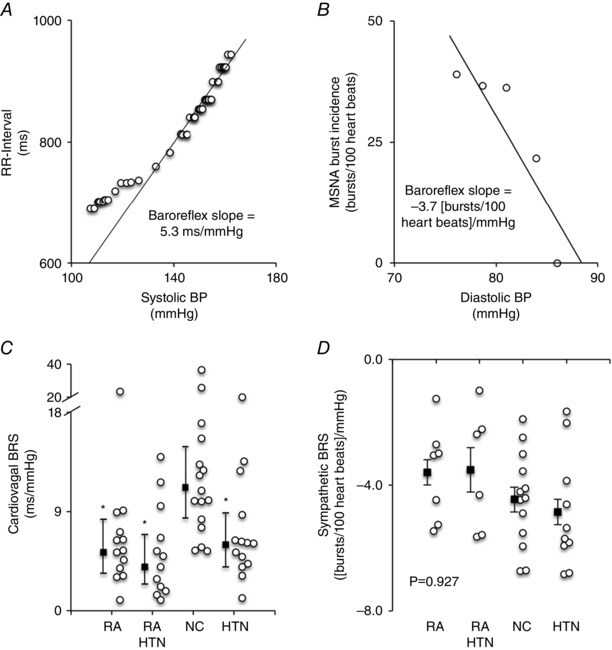
Cardiovagal and arterial sympathetic baroreflex senstivity Scatter plots from an original record of an RA patient demonstrating the relationship between RR‐interval and systolic BP (*A*), as well as between MSNA burst incidence and diastolic BP (*B*). Box and whisker plots showing cardiovagal baroreflex sensitivity (*C*), geometric mean and 95% confidence intervals) and baroreflex control of MSNA (*D*) (mean ± SEM) in the RA, RA‐HTN, NC and HTN groups. Group (black squares) and individual (white circles) data are shown. Overall effect: *P < *0.05. *Post hoc*: ^*^ *P < *0.05 *vs*. NC. *A*, RA, *n = *13; RA‐HTN, *n = *17; NC, *n = *17; HTN, *n = *16. *B*, RA, *n = *6; RA‐HTN, *n = *5; NC, *n = *9; HTN, *n = *7.

Cardiac BRS was lower in the RA, RA‐HTN and HTN groups compared to the NC group (*P = *0.002), whereas arterial baroreflex control of MSNA was not different between all groups (*P = *0.927) (Fig. 3).

### Inflammation and pain

**Figure 4:**
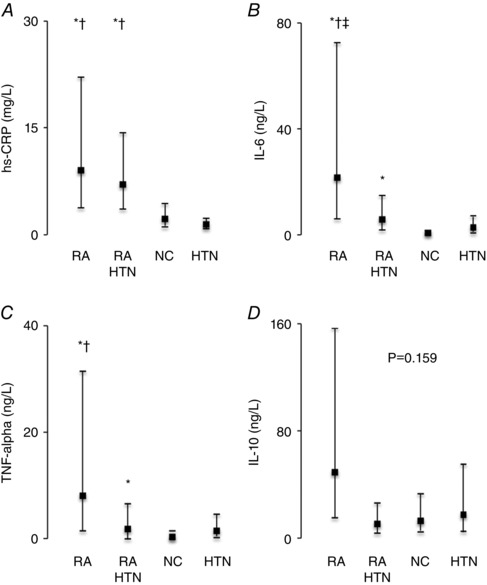
Inflammatory biomarkers Box and whisker plots showing concentrations (geometric mean and 95% confidence intervals) of hs‐CRP (*A*), IL‐6 (*B*), TNF‐α (*C*) and IL‐10 (*D*) in the RA, RA‐HTN, NC and HTN groups. Group data are shown. Overall effect: *P < *0.05. *Post hoc*: ^*^ *P < *0.05 *vs*. NC, ^†^ *P < *0.05 *vs*. HTN, ^‡^ *P < *0.05 *vs*. RA‐HTN. RA, *n = *13; RA‐HTN, *n = *17; NC, *n = *17; HTN, *n = *16.

The RA and RA‐HTN groups had higher hs‐CRP compared to the NC and HTN groups (*P < *0.001) (Fig. 4) with no significant difference between the RA and RA‐HTN groups. IL‐6 was higher in the RA group compared to the RA‐HTN, NC and HTN groups, and was higher in the RA‐HTN group compared to the NC group (*P < *0.001). The RA group had higher TNF‐α compared to the NC and HTN groups (ANOVA, *P = *0.027; *post hoc* RA *vs*. NC and RA *vs*. HTN, *P < *0.05) and numerically (but not statistically significantly) higher TNF‐α than the RA‐HTN group (*post hoc* RA *vs*. RA‐HTN, *P = *0.062). IL‐10 tended to be higher in the RA group compared to the other groups (*P = *0.159).

The RA and RA‐HTN groups had more pain than the NC and HTN groups (as measured by VAS) and the RA group had more pain than the RA‐HTN groups (RA geometric mean 37, 95% CI 22–62; RA‐HTN 13, 5–30; NC 1, 0–2; HTN 1, 0–3; *P < *0.001).

### Associations between inflammation and autonomic function

Inflammatory cytokines (IL‐6, TNF‐α and IL‐10) were positively associated with each other, whereas hs‐CRP was only associated with IL‐6 (Table 3). Both hs‐CRP and IL‐6 were positively associated with HR, although TNF‐α and IL‐10 were not. MSNA burst frequency was positively associated with hs‐CRP but not with inflammatory cytokines; however, this association was not evident when MSNA was adjusted for HR (MSNA burst incidence). Cardiac BRS was inversely associated with inflammation (hs‐CRP, IL‐6 and TNF‐α), whereas arterial baroreflex control of MSNA was not.

**Table 3:** Correlation between inflammation, pain and autonomic function

	VAS	hs‐CRP	IL‐6	TNF‐α	IL‐10
hs‐CRP	0.586*				
	<0.001	–	–	–	–
	57				
IL‐6	0.519*	0.339*			
	<0.001	0.010	–	–	–
	62	57			
TNF‐α	0.306*	0.010	0.753*		
	0.016	0.939	<0.001	–	–
	62	57	62		
IL‐10	0.114	–0.068	0.537*	0.612*	
	0.378	0.616	<0.001	<0.001	–
	62	57	62	62	
Heart rate	0.464*	0.362*	0.339*	0.059	−0.068
	<0.001	0.006	0.010	0.646	0.616
	63	57	57	57	57
Mean blood pressure	0.224	0.253	0.073	0.025	−0.138
	0.077	0.057	0.572	0.844	0.286
	63	57	62	62	62
MSNA burst frequency	0.238	0.418*	0.266	0.039	−0.044
	0.150	0.011	0.107	0.816	0.794
	38	36	38	38	38
MSNA burst incidence	−0.032	0.193	0.039	−0.033	−0.109
	0.849	0.260	0.815	0.844	0.516
	38	36	38	38	38
cBRS	−0.506*	−0.332*	−0.408*	−0.322*	−0.080
	<0.001	0.019	0.002	0.016	0.561
	55	50	55	55	55

Spearman's correlation. Values are expressed as Spearman's rho, *P* value and *n*.

^*^ Significance: *P < *0.05.

Table 4 shows the association between inflammation markers and autonomic function before and after multivariable adjustment. Following multivariable adjustment (RA, presence of hypertension, age, sex, BMI, haemoglobin), hs‐CRP remained positively associated with HR (adjusted *r* ^2^ = 0.375, *P < *0.001), whereas the associations with MSNA burst frequency and cardiac BRS were no longer statistically significant. Similarly, the associations between inflammatory cytokines and autonomic parameters disappeared following multivariable adjustment. In patients with RA, disease activity (DAS28‐CRP) was independently associated with HR (adjusted *r* ^2^ = 0.204, *P = *0.034) after adjustment for multiple variables (age, sex, BMI, haemoglobin concentration, presence of hypertension and RA duration).

**Table 4:** Association between inflammation (hs‐CRP, IL‐6, TNF‐α and IL‐10), pain (VAS) and autonomic function before and after multivariable adjustment

		Univariable^a^		Multivariable^c^		
	*N*	Rho	*P*	*r* ^2^	*F*	*P*
Dependent variable: MSNA burst frequency						
hs‐CRP	36	0.418	0.011	0.222	1.452	0.238
VAS	38	0.238	0.150	0.345	7.237	0.012*
Dependent variable: cBRS						
hs‐CRP	50	−0.332	0.019	0.364	0.683	0.413
IL‐6	55	−0.408	0.002	0.364	0.039	0.845
TNF‐α	55	−0.322	0.016	0.364	0.055	0.815
VAS	55	−0.506	<0.001	0.417	4.279	0.044*
Dependent variable: HR						
hs‐CRP	62	0.362	0.006	0.366	13.705	0.001*
IL‐6	62	0.339	0.010	0.208	0.230	0.634
DAS28‐CRP^d^	29	0.499^b^	0.006	0.204	5.123	0.034*
VAS	63	0.464	<0.001	0.526	36.661	<0.001*

a Spearman's rank.

b Pearson's correlation.

c After adjustment for age, sex, BMI, presence of hypertension, RA diagnosis and haemoglobin concentration.

d After adjustment for age, sex, BMI, presence of hypertension, haemoglobin concentration and RA duration.

DAS, disease activity score.

^*^ *P* < 0.05.

### Associations between pain and autonomic function

VAS was independently associated with MSNA burst frequency (positively, *P = *0.012), cardiac BRS (inversely, *P = *0.044), and HR (positively, *P < *0.001) after adjustment for multiple variables (RA, presence of hypertension, age, sex, BMI, haemoglobin) (Table 4).

## Discussion

In the present study, we provide the first direct evidence for heightened sympathetic outflow and reduced arterial baroreflex control of the heart in RA, whereas baroreflex control of MSNA was preserved. These autonomic alterations could occur independently of hypertension and were associated with increases in both pain and inflammation in RA. We show, in RA patients, that MSNA is heightened, whereas baroreflex control of HR is reduced, and also that this can occur independently of the presence of hypertension. We observed a significant positive but moderate association between inflammation (as measured by hs‐CRP) and MSNA.

Heightened sympathetic outflow to the heart and/or vasculature can have a multitude of deleterious consequences (Fisher & Paton, 2012) and is associated with increased mortality risk (Lown & Verrier, 1976; Cohn *et al*. 1984; Barretto *et al*. 2009). Experimental data from animals showing that inflammatory cytokines (IL‐6, TNF‐α, IL‐1β) can increase sympathetic nerve activity (Niijima *et al*. 1991; Zhang *et al*. 2003; Helwig *et al*. 2008) led to our hypothesis that an elevated circulating cytokine concentration is associated with increased MSNA in RA. By using microneurography to provide a direct assessment of sympathetic outflow, we circumvented the limitations associated with the measurement of plasma catecholamines, which reflect tissue clearance and uptake, as well as production (Esler *et al*. 1990).

Reduced cardiac BRS is present in cardiovascular diseases, such as hypertension and chronic heart failure, and predicts mortality risk following myocardial infarction (De Ferrari *et al*. 2007). Using the modified Oxford technique, which provides an assessment of BRS control across a wide BP range (Rudas *et al*. 1999), we observed that cardiac BRS was reduced in patients with RA. The underlying mechanisms may relate to altered central baroreflex modulation, as well as disruptions in afferent or efferent pathways. Nevertheless, this reduced cardiac BRS may contribute to the increased cardiovascular and mortality risk seen in RA (Maradit‐Kremers *et al*. 2005; Levy *et al*. 2008). Interestingly, the baroreflex dysfunction was specific to the cardiovagal limb because the control of MSNA was not different between groups. The prognostic significance of alterations in sympathetic BRS has not been studied; however, baroreflex activation therapy has been shown to reduce MSNA, improve symptoms and increase ejection fraction in chronic heart failure patients (Gronda *et al*. 2014). Studies of elderly individuals (Ebert *et al*. 1992) and patients with HTN (Grassi *et al*. 1998) have previously reported a differential effect on baroreflex control of the heart and MSNA similar to that observed in RA in the present study. Such observations may be attributable to distinct baroreflex pathways regulating different end‐organs. This separation may commence at the level of the primary afferent neurones and involve discreet conduits within central reflex circuits that enable the target‐organ specific control of pre‐motor and motor neurones (Polson *et al*. 2007; Simms *et al*. 2006). In addition, disparate changes in vagal and sympathetic motoneuronal modulation, and/or cholinergic signalling at the sinoatrial node, may also contribute (Rudas *et al*. 1999; Dutoit *et al*. 2010; Shantsila *et al*. 2015).

In the present study, pain was independently associated with MSNA burst frequency and cardiac BRS. These findings are in agreement with work showing that experimentally evoked chronic pain can heighten sympathetic outflow (Fazalbhoy *et al*. 2012) and reduce cardiac BRS (Duschek *et al*. 2007). Although the precise mechanisms are not fully understood, interactions between pain and cardiovascular control may be explained by an overlap in anatomical structures and pathways, including afferent pathways (e.g. baroreceptor and nociceptor projections), central modulation (e.g. nucleus of the solitary tract) and efferent pathways (e.g. descending pain inhibitory, sympathetic and parasympathetic projections) (Bruehl & Chung, 2004). In the present study, RA patients had greater self‐reported pain than RA‐HTN patients despite similar serum concentrations of hs‐CRP. This may reflect the higher pain threshold observed in hypertensive individuals (i.e. hypertensive hypoalgesia) (Ghione *et al*. 1988) or possibly an effect of the higher concentration of inflammatory cytokines in the RA group. Evidence from animal (Boettger *et al*. 2008) and human studies shows that inflammatory cytokines have a direct role in the modulation of pain perception (Hess *et al*. 2011). TNF‐α inhibition acutely blocked both central nociceptive activity and activation of the limbic system in RA patients (Hess *et al*. 2011). In RA, disease control has been shown to improve survival (Choi *et al*. 2002; Listing *et al*. 2015), although further clarification is needed to distinguish between the effects of pain and inflammation control on autonomic function.

Elevated HR is an independent predictor of mortality in the general population (Ho *et al*. 2014) and in other conditions (e.g. chronic heart failure, coronary artery disease, myocardial infarction, hypertension and diabetes mellitus) (Palatini *et al*. 2002; Fox *et al*. 2008; Ho *et al*. 2010; Hillis *et al*. 2012; Jabre *et al*. 2014). Patients with RA were observed to have an elevated HR in the present study, independent of hypertension. Aside from an increase in cardiac sympathetic nerve activity and a decrease in parasympathetic activity, there are several other potential mechanisms: chronic anaemia (Zlateva *et al*. 2010), increased metabolic rate as a result of chronic inflammation (Straub *et al*. 2010) or concomitant thyroid dysfunction (Shiroky *et al*. 1993), medication (Gilani *et al*. 2012), anxiety or depressive illness (Sheehy *et al*. 2006) and low physical activity (Sokka *et al*. 2008). Attempts to lower HR with the use of β‐blockers following myocardial infarction or in chronic heart failure has been shown to improve survival (Lopez‐Sendon *et al*. 2004). In an experimental model of arthritis, the use of carvedilol (a non‐selective β‐blocker) was associated with a reduction in markers of oxidative stress and the release of inflammatory cytokines (TNF‐α, IL‐6) (Arab & El‐Sawalhi, 2013). Whether such benefits would be manifest in RA patients treated with β‐blockers requires further study. We found that MSNA burst frequency (bursts min^–1^) was higher in RA patients compared to normotensive controls but MSNA burst incidence [bursts (100 heart beats)^–1^] was not (RA *vs*. NC, *P = *0.056; RA‐HTN *vs*. NC, *P = *0.111), probably on account of the elevated HR. Nevertheless, the observation that bursts per unit time are elevated in RA and RA‐HTN compared to NC is interpreted as being indicative of heightened sympatho‐excitation. The relatively small sample size is a potential limitation of the present study; however, this did not prevent a significantly elevated MSNA burst frequency being detected in the RA, RA‐HTN and HTN groups compared to controls. A small number of participants were smokers and, although the sympatho‐excitatory effects of smoking cannot be excluded (Hering *et al*. 2010), smoking was not statistically associated with MSNA in multivariable analysis. Notably, leg blood flow was elevated in RA and RA‐HTN groups compared to controls, possibly as a result of the vasodilatory effects of inflammatory cytokines. In animal models of sepsis, inflammatory cytokines appeared to cause vasodilatation via downregulation of α1‐adrenergic, angiotensin II and vasopressin receptors (Bucher *et al*. 2001; Bucher *et al*. 2002; Bucher *et al*. 2003). We acknowledge that substantial variability was evident in many of the parameters measured (e.g. inflammatory cytokines, HR, cBRS). All data were tested statistically for normality, and non‐normally distributed data were logarithmically transformed and parametric testing was performed. In the multivariable analysis, independent associations between pain VAS, hs‐CRP and autonomic function (HR, MSNA and cBRS) were weak to moderate and only explained between 35% and 53% of the variance. Further studies are required to determine the additional factors that may contribute to autonomic dysfunction in RA.

An important strength of the design of the present study is the inclusion of hypertensive control groups, allowing us to control for the presence of hypertension in RA and to control for the effects of medications. Athough increased MSNA and reduced cardiac BRS are known to be present in hypertension (Carthy, 2014), we have shown that, in RA, these autonomic alterations can occur independently of hypertension, suggesting an alternative mechanism (e.g. cortisol‐induced hypertension). Glucocorticoid use was higher in the RA‐HTN group compared to the RA group and may partly account for the difference in the inflammatory profile and BP seen in the present study. Glucocorticoids have been shown to reduce MSNA (Lenders *et al*. 1995), which may have attenuated sympatho‐excitation in the RA‐HTN group. Mechanistic links between immunity and hypertension have also been described, where T‐cells, macrophages and monocytes accumulate within the kidney and blood vessel wall, releasing cytokines that deleteriously affect renal and vascular function (McMaster *et al*. 2015). The cross‐sectional design precludes the establishment of causality; however, we suggest that heightened sympathetic outflow can occur prior to the development of hypertension and may possibly contribute to the development of hypertension in some patients with RA. Autonomic dysfunction may be a causal factor in the pathogenesis of RA or a consequence of the disease, or both. Future randomized controlled interventional studies are needed to clarify whether autonomic dysfunction is a cause or consequence of RA and, in particular, to differentiate between the contributions of inflammation and pain to autonomic dysfunction in RA.

In conclusion, the present study is the first to demonstrate heightened MSNA in RA. Heightened sympathetic outflow and reduced baroreflex control of HR in RA may potentially contribute to the recognized increase in cardiovascular risk. We found that, compared to inflammation, pain was more strongly correlated with MSNA (positively) and cBRS (negatively), suggesting a potential benefit in optimizing pain management to restore autonomic balance in RA. Such findings may mean that therapeutic interventions to reduce inflammation and pain in RA can restore autonomic balance and hence improve morbidity and mortality, whereas therapies that target the autonomic nervous system (e.g. sympatholytic agents, baroreflex activation) may reduce inflammation and/or pain. Interventional studies designed to evaluate these possibilities will help improve our understanding of the pathophysiology of RA and, in particular, will shed light on the interplay between inflammation, pain and the autonomic nervous system.

## Additional information

### Conflicts of interests/competing interests

The authors declare that they have no competing interests.

### Author contributions

JPF, GDK, GYL and JFRP were involved in conception of the work and critical review. AMA and JPF were involved in acquisition, analysis and interpretation of the work. AMA drafted and revised the work. All authors have approved the final manuscript and agree to be accountable for all aspects of the work in ensuring that questions related to the accuracy or integrity of any part of the work are appropriate. All persons designated as authors qualify for authorship and all those who qualify for authorship are listed.

### Funding

This study was supported by a grant from Arthritis Research UK (grant number 196633). JFRP is funded by the British Heart Foundation.
